# First report of canine ocular thelaziosis by *Thelazia callipaeda* in Portugal

**DOI:** 10.1186/1756-3305-5-124

**Published:** 2012-06-21

**Authors:** Lisete Vieira, Filipa T Rodrigues, Álvaro Costa, Duarte Diz-Lopes, João Machado, Teresa Coutinho, Joana Tuna, Maria Stefania Latrofa, Luís Cardoso, Domenico Otranto

**Affiliations:** 1 Os Bichos Veterinary Clinic, Chaves, Portugal; 2 Dr. Duarte Diz-Lopes Veterinary Clinic, Bragança, Portugal; 3 Veterinary Teaching Hospital, University of Trás-os-Montes e Alto Douro, Vila Real, Portugal; 4 Department of Veterinary Sciences, University of Trás-os-Montes e Alto Douro, Vila Real, Portugal; 5 Department of Veterinary Public Health, Faculty of Veterinary Medicine, University of Bari, Bari, Italy; 6 Parasite Disease Group, Instituto de Biologia Molecular e Celular, Universidade do Porto, Oporto, Portugal

## Abstract

**Background:**

*Thelazia callipaeda* eyeworms are transmitted by the non-biting insect vector *Phortica variegata* in Europe and infest the conjunctiva(s) of several mammalians, including dogs and humans. Infested hosts might remain asymptomatic or display clinical manifestations characterized by variable degrees of severity.

**Methods:**

From July to November 2011, nine dogs were detected with eyeworms at two veterinary clinics in Chaves and Bragança (North of Portugal). Nematodes collected from dogs were morphologically and molecularly characterized at species level.

**Results:**

Nematodes were identified as *T. callipaeda*. The number of worms collected from each dog ranged from three to 76 (average = 17.9 ± 26.8) and was not associated with the severity of clinical signs. Ocular discharge and conjunctivitis were observed in all dogs and ocular pruritus occurred in six of them. Polymerase chain reaction and sequencing of a portion of target cytochrome *c* oxidase subunit 1 gene further identified all nematodes as haplotype 1.

**Conclusions:**

This is the first report of *T. callipaeda* and associated ocular disease in dogs from Portugal, suggesting that thelaziosis should be included in the differential diagnosis of canine ocular affections. The risk of the infestation spreading from Spain and France to Portugal, through domestic dogs or wild mammals, is realistic.

## Background

Several species of *Thelazia* parasitic nematodes (Spirurida, Thelaziidae) have been reported to infest cattle and/or equids (e.g., *Thelazia gulosaThelazia lachrymalisThelazia rhodesi* and *Thelazia skrjabini*), domestic and wild carnivores, as well as humans, worldwide (e.g., *Thelazia californiensisThelazia callipaeda*) [1]. While the occurrence of *T. californiensis* is limited to western areas of the United States of America [2], *T. callipaeda* is widely distributed in far eastern countries and throughout Europe. These nematodes are transmitted by non-biting insect vectors and can be found in the conjunctiva(s) of vertebrate hosts [1].

A fruitfly species of the subfamily Steganinae, i.e. *Phortica variegata*, is the recognized intermediate host of *T. callipaeda* in Europe [3,4]. This insect transmits *T. callipaeda* third-stage larvae while feeding on ocular secretions around the eyes of a vertebrate receptive host (e.g. humans and other mammals, such as dogs and cats). Both the larvae and adults are involved in the pathogenesis of ocular disease caused by *T. callipaeda*, which is characterized by clinical manifestations ranging from ocular pruritus, lacrimation, congestion and discharge, epiphora, exudative conjunctivitis, corneal edema to keratitis and corneal ulceration in severe cases [1,5]. Treatment protocols include (i) mechanical removal of worms and (ii) administration of macrocyclic lactones, including ivermectin, milbemycin oxime and moxidectin [6,7].

For its original distribution (former Soviet republics, India, Thailand, China and Japan), *T. callipaeda* was commonly referred to as the “oriental eyeworm” [2]. However, over the last decade, infestations by *T. callipaeda* have been reported in dogs, humans, cats, foxes, rabbits and/or wolves in Italy [8,9], Germany [10], France [11,12], Switzerland [13] and Spain [14]. A thorough molecular examination of the nucleotide sequence of the mitochondrion cytochrome oxidase subunit 1 (*cox*1) gene revealed the existence of seven distinct haplotypes of *T. callipaeda* in Asia, but only haplotype 1 (h1) and another yet suggested novel unnamed haplotype in Europe [10,15]. The evidence of one unique haplotype circulating in Europe, irrespective of the several host species from which the nematodes had been collected, led to the hypothesis that the populations of *T. callipaeda* in Europe are strictly associated with the species of arthropod vector [15].

Climatic changes, including global warming, together with the increased movement of domestic animals across European countries, are facilitating the spread of several vector-borne pathogens in previously naive countries, thus increasing the risk of infections in animals and humans [16]. Current methods for disease mapping have identified large areas of Europe which are characterized by suitable climatic conditions for the development of both *P. variegata* and *T. callipaeda*[4].

This report describes the first occurrence of ocular thelaziosis by *T. callipaeda* in Portugal and provides hypotheses on the mechanisms that are leading to an increment of reports of this parasitosis and possible risks for public health.

## Methods

### Animals and samples

**Figure 1:**
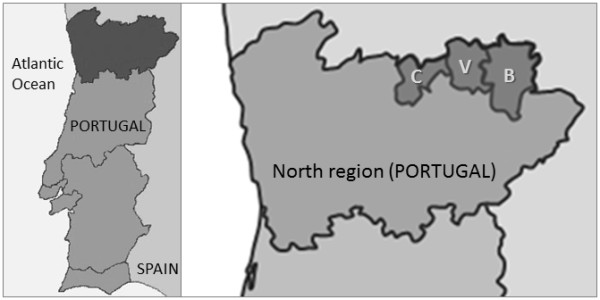
**Geographical areas in Portugal from where cases were detected.** Dogs found infested with *Thelazia callipaeda* lived in the contiguous municipalities of Chaves (C), Vinhais (V) and Bragança (B).

**Figure 2:**
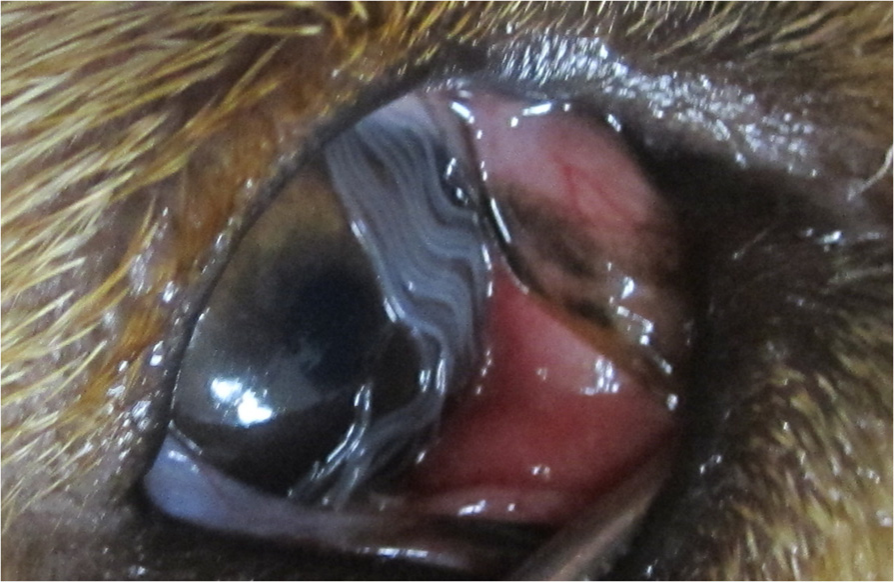
**Dog’s eye with worms.** Several *Thelazia callipaeda* nematodes in the conjunctiva of a dog (case no. 9).

From July to November 2011, nine dogs were detected with worms in their conjunctivas at two veterinary medical centers in the cities of Chaves (n = 6) and Bragança (n = 3), (North of Portugal). These dogs lived in a geographical area of ~2,460 km^2^ consisting of the contiguous municipalities of Chaves, Vinhais and Bragança (Figure 1) and had never travelled abroad. All animals were in good body condition but were referred to the practice due to an ocular discharge. After administration of an ocular anaesthetic (oxibupocraine hydrochloride, Anestocil®), an ophthalmic examination was performed and worms were collected from the conjunctivas using sterile cotton swabs or flushing with saline solution (NaCl 0.9 %) (Figure 2). Data on breed, gender, age and habitat were recorded for each animal (Table 1).

**Table 1:** Data on the identification of the dogs (n = 9) and parasite counts

**Dogs**						**Worms**
Case	Breed	Gender	Age (years)	Municipality	Habitat	F:M (n)
1	Portuguese Podengo	M	5	Chaves	Out (A, C, Pr)	6:1
2	Crossbreed	M	2	Chaves	In/Out (A, V)	4:2
3	Transmontano Mastiff	M	10	Bragança	Out (ND)	22:3
4	Labrador Retriever	M	2	Chaves	In/Out (A, Pc, Pr)	3:1
5	Labrador Retriever	M	3	Chaves	In/Out (A, Pc, Pr)	4:0
6	Portuguese Podengo	F	5	Vinhais	Out (A, Pr)	3:0
7	Labrador Retriever	F	10	Bragança	Out (ND)	41:19
8	Transmontano Mastiff	M	9	Chaves	Out (ND)	51:25
9	Crossbreed	F	3	Chaves	Out (ND)	23:6

A: apple trees; C: cherry trees; F: female; In: indoors; M: male; ND: not defined; Out: outdoors; Pc: peach trees; Pr: pear trees; V: vineyards.

### Parasite identification

Worms collected from each individual dog were morphologically identified to species and gender according to keys proposed by Otranto *et al*. [17]. Gender was assigned based on the position of the vulva anterior to the oesophagus-intestinal junction (females) and presence of five pairs of postcloacal papillae (males) [18]. Worms were then counted (Table 1) and stored in 70 % ethanol until molecular analyses. Parasite samples were subjected to specific PCR amplification of a portion (689 bp) of the *cox*1 gene [15]. Amplicon sequences were determined in both directions (using the same primers individually as for the PCR) by visual inspection of the individual electropherograms. Sequences were aligned using the ClustalX program [19] and the alignments were compared with sequences available in public databases for the *cox*1 of *T. callipaeda*.

## Results

Table 1 presents data on the identification of the dogs and number of parasites recovered. Affected dog breeds/crosses were Labrador Retriever, crossbreeds, “Podengo Português” (Portuguese Podengo) and “Cão de Gado Transmontano” (Transmontano Mastiff), aged from 2 to 10 years, both females and males. Besides ocular discharge, conjunctivitis was observed in all dogs, and ocular pruritus in six of them (cases nos. 1, 2, 4, 5, 8 and 9). No apparent association was observed between worm burden and severity of clinical signs. The number of worms collected per dog ranged from three (all female parasites) to 76 (51 females and 25 males). The average number of *T. callipaeda* worms counted in the nine infested dogs was 17.9 (± 26.8 SD). The morphological identification was confirmed by specific molecular identification and only h1 nematodes were identified from all the nine dogs.

## Discussion

The present study reports the first autochthonous cases of infestation with *T. callipaeda* in dogs from Portugal. Indeed, none of the nine dogs had ever travelled abroad. Interestingly, all *T. callipaeda* nematodes were identified as h1, thus far the only characterized haplotype reported in Europe. All infested animals lived outdoors at high risk of infestation.

This study involved dogs permanently living in the municipalities of Chaves, Vinhais and Bragança, which lie on the Portuguese-Spanish border. Latitude (between 41’ 00” and 48’ 00” N), climate, altitude and vegetation are similar to those of other European countries and regions where thelaziosis is now considered endemic, e.g. Italy [20,21], France [11,12] and Spain [14]. The geographical area of Chaves, Vinhais and Bragança is characterized by a rural environment with many hunting and shepherd dogs, as well as habitats suitable for the development of *P. variegata*. This scenario is in accordance with the observations collected using a predictive geoclimatic model, which identified this area as suitable for the life cycle of the intermediate host [4]. Although the vector of *T. callipaeda* in northern Portugal is most likely *P. variegata*[4], further studies are required to confirm this hypothesis. Since the nine affected dogs had never travelled abroad, it is presumed that the infestations were of autochthonous origin.

In the Spanish provinces of Orense and Salamanca, 135 and nine cases of canine thelaziosis have been described, respectively [22]. The fact that these provinces are contiguous with the North of Portugal (Orense to the north and Salamanca to the east) supports the hypothesis that this infestation might also be emerging in this country. The existence of a sylvatic life cycle in wild animals, such as foxes and wolves, could be responsible for the spread of the parasite across Europeans countries, because these animals can freely roam through borders and act as reservoirs for this nematode [1]. Under these circumstances, the risk of a spread of the infestation from Spain to Portugal is realistic, for instance through domestic dogs or wild mammals. Moreover, during summer months, a large number of emigrants return home for vacations with their pets from regions of southern and central France where ocular thelaziosis is endemic [11,12]. In association with the spread of the infestation from Spain, this could provide an additional explanation for the emergence of *T. callipaeda* in Portugal.

Infestations with *T. callipaeda* are spreading across Europe and becoming a serious problem in several countries. In Italy, *T. callipaeda* infests around 60 % of dogs in Potenza, Basilicata [8]. In the region of La Vera, Spain, the prevalence of infestation reached almost 40 % in the canine population [14]. Being limited to clinically suspect cases referred to the two veterinary centers, our report does not allow an estimation of the prevalence of *T. callipaeda* infestation in the Portuguese canine population to be made. Nevertheless, the number of clinical cases of canine thelaziosis diagnosed from July to November 2011 in the two veterinary clinics involved in this study (i.e. 16 clinical cases out of 2300 dogs brought in for general veterinary consultation) allows an approximate estimation of disease prevalence (i.e. 0.7 %). This projection includes seven additional cases of canine thelaziosis not described in the Methods, Results and Table 1, which were diagnosed only based on clinical presentation and morphological identification of the nematodes collected (unpublished results).

The low estimated prevalence of infestation detected in the present study might be due to the fact that only dogs presenting clinical signs were examined. Thus, it is possible that a considerable number of asymptomatic dogs are also infested. A recent introduction of *T. callipaeda* in Portugal may also account for this estimated low prevalence. The investigation in the region of La Vera revealed that the proportion of infested dogs presenting clinical signs (~15 %) was considerably lower than that recorded in apparently healthy infested dogs (~85 %) [14]. Under laboratory conditions the transplantation of adult worms into rabbit eyes induced a severe inflammatory response 3–5 hours post-infestation; however, the clinical signs gradually diminished after one week [23]. These findings might contribute to explaining the high number of asymptomatic but infested animals observed in some studies and a similar scenario may also occur in the North of Portugal.

Up until now, there have only been four reported cases of human infestation with *T. callipaeda* in Europe, two from Italy and two from France [24]. However, in the last two decades the number of cases of human ocular thelaziosis by *T. callipaeda* in Asian countries has increased [5]. Therefore, it is possible that the number of humans infested in Europe may rise in the future. Due to the potential risk of human infestation, an improved awareness of veterinarians and medical ophthalmologists for this parasite is warranted. In order to control zoonotic eyeworm infestation, correct diagnosis and treatment of infestations in domestic animals are crucial. The administration of broad spectrum macrocyclic lactones on a regular basis as a preventative measure against canine thelaziosis is advisable [18].

## Conclusions

The present report provides the first description of *T. callipaeda* in dogs from Portugal. Thelaziosis should be included in the list of differential diagnoses in dogs with ocular manifestations. Epidemiological studies of *T. callipaeda* infestation in dog populations, including both clinically affected and asymptomatic animals, are needed in the northern as well as in the southern regions of Portugal. Addressing these gaps in knowledge will assist the definition of endemic areas and allow the establishment of more effective prophylactic and therapeutic measures against ocular thelaziosis.

## Competing interests

The authors declare that they have no competing interests.

## Authors’ contributions

Collected and characterized clinical samples: LV, FTR, AC, DD-L, JM and JT. Coordinated the study: LC. Performed morphological analysis: TC and MSL. Performed molecular analysis: MSL and DO. Analyzed data, drafted and revised the manuscript: LV, FTR, LC and DO. All authors read and approved the final version of the manuscript.
